# Gender Recognition from Unconstrained and Articulated Human Body

**DOI:** 10.1155/2014/513240

**Published:** 2014-04-07

**Authors:** Qin Wu, Guodong Guo

**Affiliations:** ^1^Department of Computer Science, Jiangnan University, Wuxi, Jiangsu 214122, China; ^2^Lane Department of Computer Science and Electrical Engineering, West Virginia University, Morgantown, WV 26506, USA

## Abstract

Gender recognition has many useful applications, ranging from business intelligence to image search and social activity analysis. Traditional research on gender recognition focuses on face images in a constrained environment. This paper proposes a method for gender recognition in articulated human body images acquired from an unconstrained environment in the real world. A systematic study of some critical issues in body-based gender recognition, such as which body parts are informative, how many body parts are needed to combine together, and what representations are good for articulated body-based gender recognition, is also presented. This paper also pursues data fusion schemes and efficient feature dimensionality reduction based on the partial least squares estimation. Extensive experiments are performed on two unconstrained databases which have not been explored before for gender recognition.

## 1. Introduction

Gender or sex is an important clue about a human being. Humans are divided into two groups: male and female. Different gender groups may present different habits. For example, boys and girls may like different toys; men and women may prefer different clothes (e.g., color, style, pattern, etc.). In business intelligence, knowing the gender (and/or age) of the customers may help business managers to advertise their products according to different customer groups and collect valuable demographic information about the customers, for example, how many women entering a retail store or a shopping mall within a given period of time. In security, the gender information may be used for access control, for example, restricting access to certain areas based on gender. In image retrieval, gender (and/or age) can be a useful semantic concept for photo organization and search, for example, finding men or women in certain scenes. In social interactions or activities, males and females may perform differently and have different roles. Therefore recognizing the gender of a person has broad applications.

Humans have the capability to perceive the gender of each other. It has been an active research in psychology to study the perception of gender by humans [1–3]. In psychology studies, the stimulus is usually the face photos for gender perception. In computational visual analysis for gender classification or recognition, most of the existing works used face images. For example, see [4–8].

Recently, some approaches, for example, [9, 10], showed that human bodies can be used for gender classification. Successful results are shown on pedestrian images where a manually drawn box containing a pedestrian is used globally for body information extraction.

The advantages of using body over face images for gender recognition include the following. (1)* Image Resolution.* When the face region has a low resolution, or is motion blurred, the facial features might not be usable for gender classification. However, the body image may still be useful for separating males from females. (2)* Viewpoint Change.* When the head pose is very different from frontal views, the face-based gender recognition might have low performance or even not be applicable. However, the body image can still be used. Even the back view of the body can be used for gender recognition [9, 10]. (3)* Acquisition Distance.* When the camera is far away from the person, the face image will not provide sufficient information for gender discrimination. However, the body image may still be usable. (4)* Occlusion.* When the facial part is occluded, the face image might not be used to extract gender information. However, the body image is still useful, even when some body parts are occluded.

**Figure 1:**
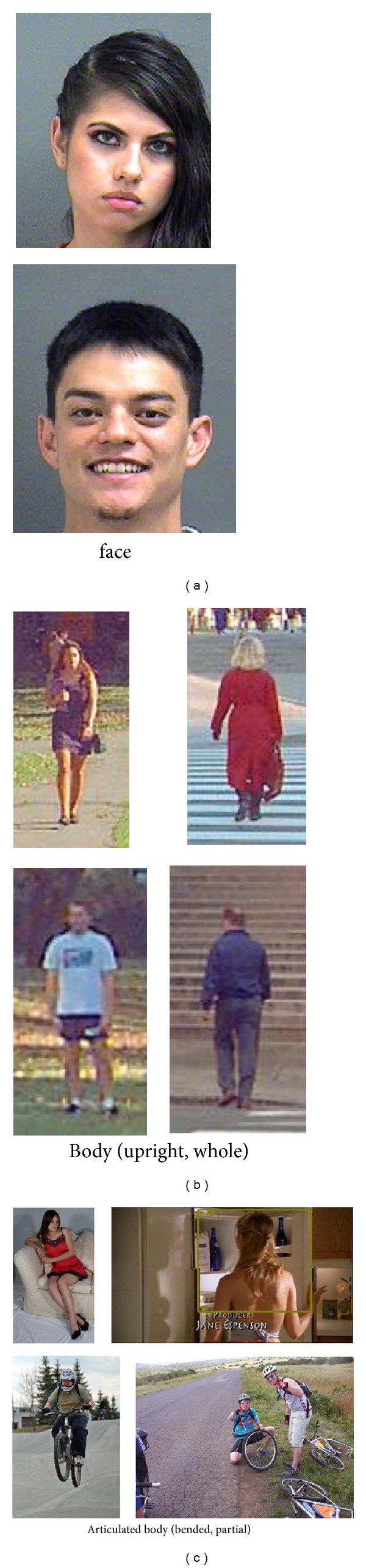
Gender recognition using different modalities. (a) Face images; (b) upright and whole body, for example, pedestrian, where the alignment problem is trivial; (c) articulated bodies which have various body part variations and even only partial body being visible.

However, in those pioneering approaches to gender recognition from body, for example, [9, 10], only the upright body images with the whole body appearance are investigated. The data typically used are pedestrian images, such as the MIT pedestrian database [11] which is a common database for pedestrian detection. One important requirement is that the whole body in upright appears in each image, as shown in Figure 1(b), and thus there is no need to worry about aligning the body images in matching. Features can be extracted from the whole body images and used to train a classifier.

In reality, human bodies may undergo various articulations, bending, rotations, movements, and interactions with objects. Furthermore, an articulated body may be viewed by a camera just partially in a typical image. All these realistic variations, as shown in Figure 1(c), make the body appearance change significantly. But human viewers can perceive gender from those articulated bodies without any difficulty. This motivates us to explore a computational method to recognize gender from the articulated body. It is certainly a great challenge. Please note that some of the example images in Figure 1(c) have no face (a back view) or heavily occluded faces, but human viewers can still perceive the gender. So we believe that some cues from the body other than the face are used by humans to recognize gender.

Furthermore, humans can perceive gender from part of a body, not necessarily using the whole body. This motivates us to think about a new issue: gender recognition may use partial body rather than the whole body.

It is well known that men and women do have significant biological differences in their bodies. Men and women may wear different clothes (color, style, and patterns). These evidences support the body-based discrimination between males and females.

This work studies the following two problems:

- whether gender recognition can be performed or not in articulated body images using a computational method,
- how to extract gender information from an articulated body.

The major contributions of this paper include

1. presenting a new problem called* articulated body-based gender recognition*; to our best knowledge, this is the first time to study the gender recognition problem using articulated human body parts;
2. developing a method to recognize gender from an articulated body;
3. a systematic study of some critical issues in body-based gender recognition, such as how informative each body part is, how many body parts are needed, what are good representations of body parts, and how accurate the gender recognition system can achieve in challenging, unconstrained, real-world images.

The remaining of the paper is organized as follows.  Section 2 describes the proposed gender recognition method based on articulated body parts.  Section 3 presents the experiments and discussions. Finally, conclusions are drawn.

## 2. Articulated Body-Based Gender Recognition

**Figure 2:**
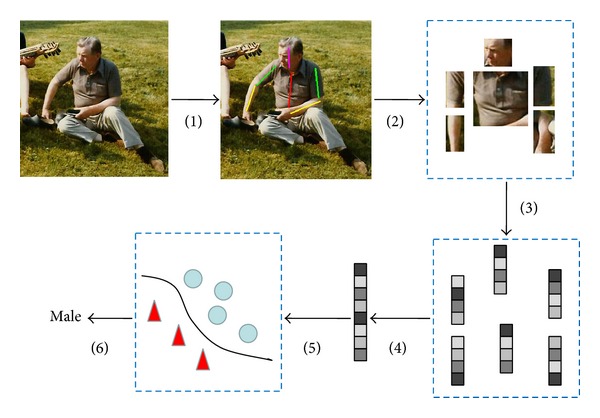
The proposed framework for gender recognition from articulated body.

To recognize gender from the articulated body, this paper proposes detecting the body parts first. Then the detected body parts are normalized into standard sizes and orientation. Various feature descriptions can be used on each body part and combined together to execute gender recognition. A simple illustration of the proposed approach is shown in Figure 2.

A general paradigm in object recognition is to take the example-based-learning approach. That is to say, some example images of each object class have to be collected in the learning stage in order to learn a classification function and then apply it to testing examples. For the problem of gender recognition from an articulated body, this work also chooses the procedure of example-based learning.

In this section, we will present how to deal with the articulated body and partial views, discuss how many body parts to use, introduce the part representations, and describe data fusion schemes for the gender recognition problem. The PLS method is also described.

### 2.1. Articulated Body Parts Detection and Normalization

**Figure 3:**
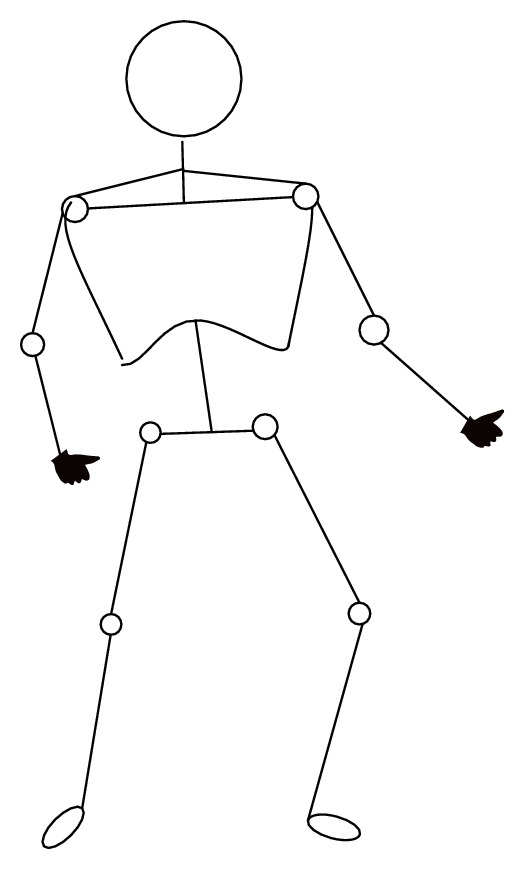
An illustration of the many degrees of freedom for articulation in a human body.

As illustrated by the joints in Figure 3, the human body has many degrees of freedom to change its shape and perform different actions (e.g., in various sports games and even daily life activities). Thus, there exist many possible variations in an articulated body, such as upper and lower arms rotations, head movements, torso bending, and leg kicking. Further, body parts can occlude each other, or be occluded by other objects, depending on the viewpoints. All these factors have to be considered in gender recognition from an articulated body. Body parts detection itself is a very challenging problem. Fortunately, recent research has made significant progress in body detection and body part localization [12–14].

To employ a set of articulated bodies for visual learning of gender, the first and most important thing is how to align the images of articulated bodies. This is very critical in developing a system for gender recognition from articulated bodies. One cannot simply extract features from the given images without considering the alignment problem. As shown in Figure 1(c) it is not trivial to align the articulated bodies with various body pose variations and occlusions.

In order to align human bodies with various poses, we perform human body detection and body parts detection first. Then we* “normalize”* the detected body parts, for example, torsos or upper arms, from different individuals into the same location and size in a common two-dimensional Cartesian coordinate system.

Another issue in body-based gender recognition in real-world images is that sometimes only partial human bodies are visible in the image frame, or some body parts are heavily occluded by other objects. To deal with “partial views” of human bodies, only the upper body is used for gender recognition in this study. Actually, the upper body conveys the major information for human perception of gender. Although the lower body may contain some information when visible, this work does not use it in order to make our system general and robust to deal with many possible situations in reality. In fact, the lower bodies are frequently occluded by other objects or do not appear in the camera field of view (FOV) in many real-world images, but it will not be a problem for our system.

In the following, some simple description about upper-body and part detection and normalization will be presented.

#### 2.1.1. Upper-Body Detection and Part Localization

In movies, TV shows, or consumer photos, many times only the upper body is visible in the camera field of view. Thus detecting the upper body is more general and robust than the whole body detection. The scheme of using a sliding window followed by nonmaximum suppression is usually adopted for upper-body detection [13, 14]. In our approach, the part-based model [13] is adopted, since it can do an excellent job in upper-body detection.

Then an image segmentation can be performed using the Grabcut method [15] to separate the whole upper body from the background, and an inference about the local body part can be executed based on the segmentation, as suggested by Eichner et al. in [14]. The key idea of the body part localization is to use a pictorial structure model [12], which models the posterior of a configuration of parts *L* = {*l*_*i*_} in an image *I*:

(1) $$\begin{array}{c} P\left(L\;|\;I,\Theta \right)\propto \exp\left(\underset{\left(i,j\right)\in E}{\sum }f\left(l_{i},l_{j}\right)+\underset{i}{\sum }g\left(I\;|\;l_{i},\Theta \right)\right), \end{array}$$

where the unary potential *g* is about the local image likelihood for a part in a particular position, the pairwise potential *f* is to model the prior about the relative position of parts, and Θ denotes the parameters of the model.

**Figure 4:**
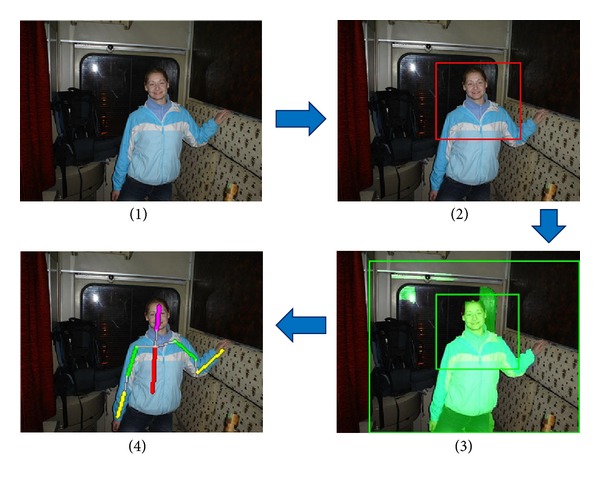
Main steps of upper-body detection and body parts localization. (1) Input image, (2) upper-body detection, (3) segmentation of the whole upper body from background, and (4) body part inference.

An illustration of the main steps of upper-body detection and local part localization is shown in Figure 4. This paper mainly focuses on how to process the body parts for gender recognition.

#### 2.1.2. Body Parts Normalization

After upper-body detection and body parts localization, the position of each body part in a two-dimensional image plane is known. However, for different images, the detected body parts may have different sizes at various scales and the body parts may have arbitrary articulations when performing distinct actions or interacting with varied objects. To learn gender from the example body parts with arbitrary articulations and match with unseen human bodies, a method has to be developed that can align the same body parts, for example, torso, but from different persons, at various scales, and with distinct articulations, into a common coordinate. We define this important procedure as* body parts normalization*. But how to perform the normalization?

**Figure 5:**
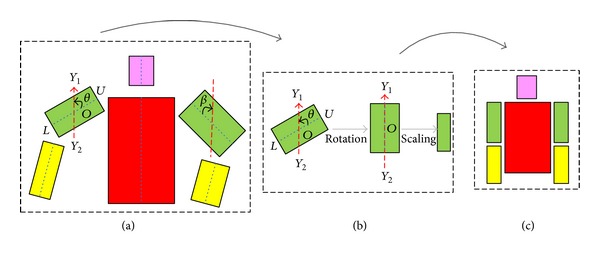
An illustration of the body parts normalization process.

First, represent each body part as a rectangular patch. Suppose a body part is centered at *O* (see left upper arm in Figure 5(a) denote the two ends of a body part as *U* (upper side) and *L* (lower side); *Y*_1_ *Y*_2_ is the line parallel to the arm and passes through the center *O*. Define the angle *θ* = *∠ROY*_1_ as rotation angle, as illustrated in Figure 5(a). If the body part is the left upper arm or the left lower arm, rotate the body part clockwise by an angle of *θ* around the center *O*. If the body part is the right upper arm or the right lower arm, rotate the body part counterclockwise by an angle of *θ* around the center *O*. After the rotation, the rectangle patch is changed to the vertical direction. By performing the in-plane rotation for each body part rectangular patch, all detected body parts have the same (vertical) orientation.

Next, deal with the scale variations due to the different viewing distances and distinct individuals. For any given image *I*_*j*_, denote the six rectangular patches of the upper body parts as *R*_*i*_(*I*_*j*_) (*i* = 1,2,…, 6). The width (denote as *w*_*i*_(*I*_*j*_)) and height (denote as *h*_*i*_(*I*_*j*_)) of the image *I*_*j*_ are known from the body parts detection. Suppose the standard width and height of the body part *i* is given as *w*_*i*_^*s*^, *h*_*i*_^*s*^; then compute the scaling factor for body part *i* as follows:

(2) $$\begin{array}{c} s_{i}^{w}\left(I_{j}\right)=\frac{w_{i}^{s}}{w_{i}\left(I_{j}\right)},\\ s_{i}^{h}=\frac{h_{i}^{s}}{h_{i}\left(I_{j}\right)}, \end{array}$$

and then apply scaling factors *s*_*i*_^*w*^(*I*_*j*_) and *s*_*i*_^*h*^(*I*_*j*_) to the body part rectangular patch *R*_*i*_(*I*_*j*_), to change it into a standard size.

The standard width *w*_*i*_^*s*^ and height *h*_*i*_^*s*^ for each body part patch can be predefined based on the human knowledge about body shapes and required image resolutions or adjusted from the image data. In our approach, we compute the mean values of each body part rectangular patch sizes:

(3) $$\begin{array}{c} w_{i}^{s}=\frac{1}{n}\overset{n}{\underset{j=1}{\sum }}w_{i}\left(I_{j}\right),\\ h_{i}^{s}=\frac{1}{n}\overset{n}{\underset{j=1}{\sum }}h_{i}\left(I_{j}\right), \end{array}$$

where *i* = 1,2,…, 6 denotes the *i*th body part and *n* is the total number of detected humans in the training set. We use the mean values as the standard sizes for each body part. Please note that different body parts may have different sizes as their standards.

In practice, the rotation and scaling are performed by image resampling. After the rotation and scale changes, the body parts normalization is completed. There is no need to perform any translations, since features are extracted from each body part, rather than from raw image patches.

### 2.2. How Many Body Parts?

Since it is for the first time to recognize gender through decomposition of the articulated body into local parts, it might be interesting to study how informative each body part is and how many body parts are needed to combine together for the purpose of gender recognition.

A key question is, do these body parts have equal contributions to gender recognition? Since no previous work has studied this problem, it is not clear to us (and other researchers) how informative each body part will be. We perform an empirical study about this using various representations (see Section 2.3) and check the results in Section 3. The basic idea is to use each single body part for learning the classifiers and testing and compare the recognition accuracies to measure the informativeness of each body part. We believe that this result is important and will provide practical guidance for body-part-based gender recognition.

Another key question is, how many body parts are needed for gender recognition? One may think that the more body parts there are to use, the higher the recognition accuracy will be. To verify whether this conjecture is true or not, an empirical study is presented by combining different body parts together based on various part representations. From our study, we found that the conjecture is not true. More details and discussion about it are shown in Section 3. As a result, one should use only the body parts that can be detected reliably and contain discriminative information and combine them together for gender recognition.

The next question is, how to combine the selected body parts. One solution is to extract features from each body part and then combine the features from all selected body parts. Another solution is to put all normalized parts into a fixed coordinate system and then extract features from the warped and aligned body parts altogether. We used the first solution in our system, since it is easier to implement and may suppress noises from the background.

### 2.3. Representation of Body Parts

After obtaining the normalized body parts, we use various operators to extract features as the representations of the body parts. Since no previous work has studied the articulated body or body-part-based gender recognition, it is unknown what kinds of features are useful and discriminative. To discover this, we predefine some features that may be useful, compare the performance of these representations, and then find what representations are good for gender recognition in articulated body images.

The following four features are extracted to represent the body parts: the histogram of oriented gradient (HOG) [16], local binary patterns (LBP) [17], scale-invariant feature transform (SIFT) [18], and RGB colors (where the histograms are computed for each color channel and concatenated). The purpose is to evaluate these features that were originally proposed for other computer vision problems and discover whether they are useful or not for our problem.

Given the representations, the support vector machine (SVM) [19] is applied to learn two-class classifiers.

### 2.4. Data Fusion

Data fusion is usually useful to improve the performance in decision making when multiple sources of information are available [20]. By combining different sources of data, a recognition system can be made more robust and more confident and even with higher accuracy. For gender recognition in unconstrained and articulated body images, various noise and uncertainties exist; for example, the body parts might be localized not accurately or occluded by other body parts or objects; the illumination may change significantly; images are highly cluttered; contrast may be poor; motion blur may be present because of camera movement or shaking; and so forth.

To make the system more robust and possibly with higher accuracy, this paper also proposes a data fusion idea and validates if data fusion could be helpful for the problem. Thanks to the multiple representations of the data (see Section 2.3), we can explore data fusion schemes by combining different representations of body parts. Two schemes are investigated. One is to combine the features together into a long feature vector and then use the SVM for training and classification, while the other is to combine the classification results of several representations to make a new decision by majority voting; that is, given the data *x*, the final decision *D*(*x*) is based on a voting from multiple classifiers, *c*_*i*_, *i* = 1,2,…, *m*. Suppose *m* is an odd number and *k* classifiers has the same decision, where *k* ≥ (*m* + 1)/2, then the decision *D*(*x*) will take the same classification decision as that of the *k* classifiers.

The two data fusion schemes, called a feature-level fusion and a decision-level fusion, respectively, are examined and compared to see which one is better.

### 2.5. Partial Least Squares Estimation

Recently, the PLS method [21, 22] has shown good performance in some computer vision problems, such as pedestrian detection [23], face recognition [24], and age estimation [25]. Inspired by this, we are interested in evaluating the performance of the PLS method in the new problem of articulated-body-based gender recognition.

In previous applications to solve computer vision problems, the PLS was used mainly for dimensionality reduction [23, 24]. Guo and Mu [25] adapted the PLS to age estimation and showed the successful use of the PLS for both feature dimensionality reduction and age regression. Actually the PLS method itself can do classification [22], which has seldom been explored in previous approaches. Here we want to evaluate the PLS's capability of both dimensionality reduction and classification.

The linear PLS algorithm [21, 22] is to model the relation between two sets of variables. Denote by *𝒳* ⊂ *ℛ* ^*N*^ an *N*-dimensional space of variables representing the first block. Similarly, denote by *𝒴* ⊂ *ℛ* ^*M*^ a space representing the second block of variables. PLS models the relations between these two blocks by means of score vectors. It decomposes the (*n* × *N*) matrix of zero-mean variables **X** and the (*n* × *M*) matrix of zero-mean variables **Y** into the form

(4) $$\begin{array}{c} X=TP^{T}+E,\\ Y=UQ^{T}+F, \end{array}$$

where **T** and **U** are (*n* × *p*) matrices of the *p* extracted score vectors (latent vectors), the (*N* × *p*) matrix **P** and the (*M* × *p*) matrix **Q** represent matrices of loadings, and the (*n* × *N*) matrix **E** and the (*n* × *M*) matrix **F** are the matrices of residuals. The PLS method, which in its classical form is based on the nonlinear iterative partial least squares (NIPALS) algorithm [26], finds weight vectors **w**, **c** such that

(5) [cov⁡(t,u)]2=[cov⁡(Xw,Yc)]2=max|r|=|s|=1[cov⁡(Xr,Ys)]2,

where cov⁡(**t**, **u**) = **t** ^*T*^ **u**/*n* denotes the sample covariance between the score vectors **t** and **u**. The NIPALS algorithm starts with random initialization of the *𝒴*-space score vector **u** and repeats a sequence of iterations until convergence [26].

In the proposed gender recognition, **X** denotes the body part representations, while **Y** is reduced to a scale value of class labels. Two issues of the PLS method will be validated:

1. how efficient for dimensionality reduction,
2. how accurate for classification (avoid using the SVMs).

## 3. Experiments and Discussions

**Figure 6:**
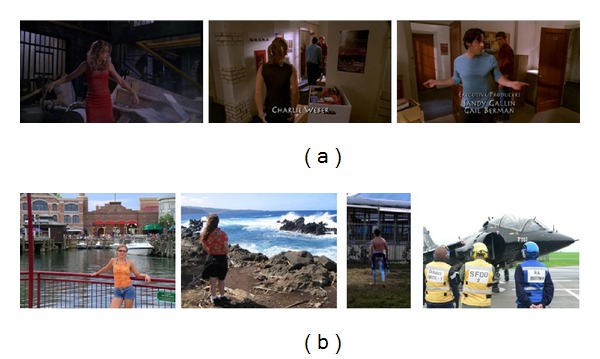
Some example images in Buffy (a) and PASCAL (b).

The proposed gender recognition method is performed on two databases that contain unconstrained and articulated human body images. One is called Buffy which contains video frames from the “Buffy: The Vampire Slayer” TV show, and the other is the PASCAL VOC 2008 challenge [27]. Both databases are publicly available [28, 29] and used for human pose estimation. But these two databases have not been evaluated for gender recognition yet. Some example images are shown in Figure 6. The annotated stickmen on both data sets are also provided with the image data. The Buffy data set contains episodes 2–6 of Buffy's season 5 for a total of 748 frames. It has TV show in uncontrolled conditions with very cluttered background, dark illumination, and persons appearing with a large range of scale changes. In pose estimation, usually episodes 3, 4 are used for training, while 2, 5, and 6 for testing [14]. We followed the same division for both body part localization and gender recognition. Notice that there are only 49 males but more females in episodes 2, 5, and 6. To avoid the very unbalanced gender distribution in reporting classification accuracies, 53 females are randomly selected from episodes 2, 5, and 6 for the experiments. The PASCAL data set consists of 538 amateur photos (317 males, 221 females) with bad illumination, low image quality, and many different articulations. The whole Buffy data set is used for training to detect the body parts on PASCAL. Both databases are challenging for human body detection and parts localization [14], while our focus is to recognize gender in those unconstrained and articulated body images. To our best knowledge, this is the first time to recognize gender from the articulated body parts and perform experiments on these two databases. And a standard fourfold cross-validation scheme is used to evaluate all results under various experimental conditions.

In order to understand the gender recognition performance in more detail, the experiments are presented in two cases: (I) the body parts are known, for example, annotated manually; (II) the body parts are unknown, but detected automatically by a method. Case I is designed to show how accurate our gender recognition methods can achieve, without the influence of incorrect body part localization. Case II is designed to show the performance of an automated system that can be developed for real applications.

### 3.1. Case I: Gender Recognition with Annotated Body Parts

To avoid possible influence of incorrect body part detection, the annotated stickmen are used to find the body parts in evaluating the gender recognition performance. We study which representations are better, how informative each body part is, and how many body parts are needed to combine together to achieve a higher recognition accuracy. And data fusion schemes are also applied to further improve the accuracy.

Given a body image and the annotated stickmen, the rectangular patch of each body part is determined as follows. For head and torso, let both the width and height of the rectangle be equal to the stickman's length. For the limb, let the height of the rectangle be the same as the stickman's length and let the width be half of the stickman's length. Then each body part is normalized into the standard size and is oriented using the method presented in Section 2.1.2. To determine the standard sizes, the mean values of the sizes of each body part are computed from the training data, and the closest integer values are used as the standard sizes. The standard sizes of each body part are different for the two databases. In Buffy, the selected standard sizes are 170 × 170 for torso, 90 × 90 for head, 100 × 50 for upper arms, and 70 × 35 for lower arms. In PASCAL, the sizes are 80 × 80 for torso, 40 × 40 for head, and 60 × 30 for both upper and lower arms. One can also see that the parts are smaller in PASCAL than Buffy. Meanwhile, each body part is rotated into the vertical direction for both the training and test body parts.

Then we represent each body part by extracting four different features, that is, HOG, LBP, SIFT, and RGB. Dense sampling with the same intervals is used for HOG, LBP, and SIFT. For RGB, the color histograms of 32 bins are computed in each color channel and concatenated into a feature vector. In the following, several important aspects in gender recognition are studied.

#### 3.1.1. Representations

The HOG, LBP, SIFT, and RGB features work well in some other problems, such as face recognition and pedestrian detection, but it is unknown whether they can be used for gender recognition from articulated bodies.

Comparisons between the four different representations on Buffy and PASCAL are shown in Tables 1 and 2, respectively. For the first four rows, one can see that the color features using RGB histograms work the best on Buffy, but the worst on PASCAL. The other three features, HOG, LBP, and SIFT, work similarly well on both databases, with the SIFT slightly worse on Buffy.

#### 3.1.2. How Informative Each Body Part Is

From the first two columns in Table 2, one can find that (both left and right) lower arms cannot separate male from female, while other body parts, such as upper arms, head, and torso, work relatively better (columns 3–6), with the representations of HOG, LBP, and SIFT. The lower arms show higher accuracies on Buffy (Table 1) than on PASCAL (Table 2), but their accuracies are still significantly lower than other body parts. So the lower arms are not informative body parts for gender recognition. Further, one can observe that using single body part is not a good strategy and not robust. So it is better to combine several body parts together based on the discriminative power for each of them.

#### 3.1.3. How Many Body Parts to Combine Together

**Figure 7:**
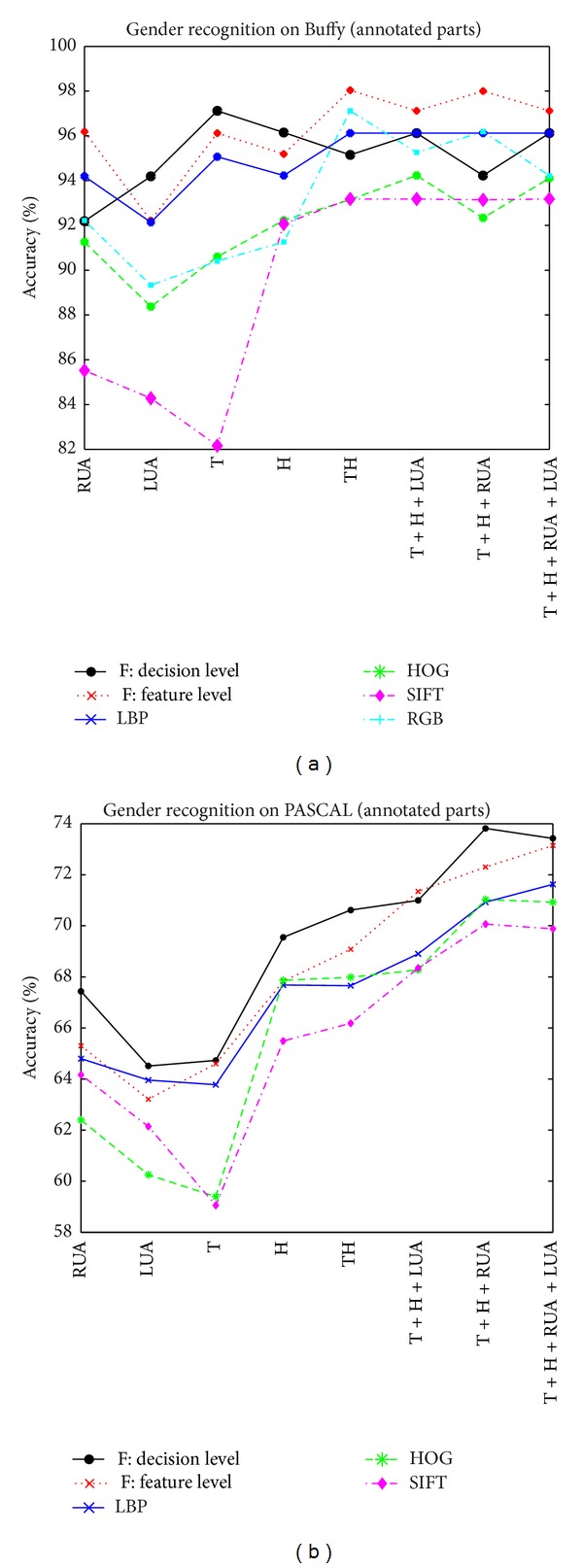
Gender recognition results with annotated parts on Buffy (a) and PASCAL (b).

Combining several body parts together will make it more robust and have a better gender recognition performance. From columns 7–10 in Table 2 or Figure 7, one can easily see the effect of combining body parts on PASCAL. Since the lower arms are not discriminative, we do not use them in part combination. Although some single body parts already have high accuracies on Buffy, combining them can still improve the recognition accuracy further (see Table 1). Interestingly, having more parts not necessarily delivers a higher accuracy. That is why we need to study which parts to combine together, rather than using all available parts. Why the gender recognition accuracy is not necessarily higher when using more body parts? There are three reasons, we think, to interpret it:

1. some body parts are not as informative as others,
2. some body parts cannot be detected reliably and accurately in practice, especially in a challenging image set,
3. some body parts might be occluded by other parts or objects more frequently than others, which causes uncertainties in gender recognition.

#### 3.1.4. Data Fusion Schemes

As stated in Section 2.4, two data fusion schemes will be evaluated. The results are shown in Tables 1 and 2 (the last two rows) and Figure 7. In Buffy, the three best features, that is, RGB, LBP, and HOG, are used for fusion. If the SIFT is added for fusion, the result becomes worse (not shown here). The feature-level fusion scheme gets the highest accuracy of 98.04% using only two body parts: head and torso. It is slightly higher than the decision-level fusion with an accuracy of 97.12% using only one body part torso, which has the same value as using the single feature of RGB on head and torso.

In PASCAL, the best three features, HOG, LBP, and SIFT, are used for data fusion. The scheme of decision-level fusion gets the highest accuracy of 73.82% on three body parts (head, torso, and right upper arm), which is slightly higher than the 73.14% accuracy using feature-level fusion on four body parts (head, torso, and both upper arms). The recognition accuracies of both fusion schemes are higher than the best of 71.62% when using single features (LBP feature with four body parts: head, torso, and upper arms; see the last column of row 2 in Table 2).

### 3.2. Case II: An Automated Gender Recognition System

In the second case, the performance of the automated gender recognition system is evaluated, which detects the upper body and related body parts automatically* without using part annotations* in either gender learning or testing. The standard fourfold cross-validation is used again to measure the performance. In Case II, the body parts might be detected imprecisely or incorrectly. But we want to verify how robust and accurate our system can achieve in automatic gender recognition.

**Figure 8:**
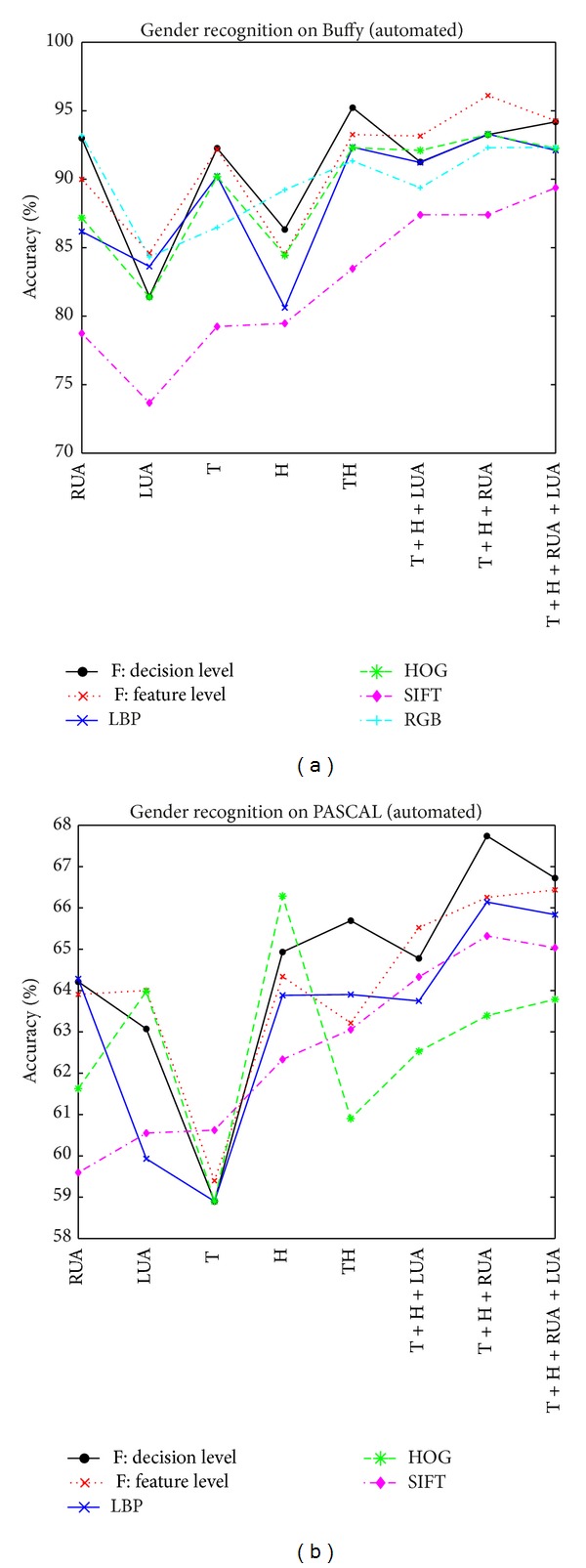
Automated gender recognition on Buffy (a) and PASCAL (b).

Similar to Case I, several important aspects in automatic gender recognition are evaluated, including body part representations, how informative each body part is, how many body parts to combine, and data fusion schemes. The experimental results are shown in Tables 3 and 4 and Figure 8.

Table 3 and Figure 8(a) show the recognition performance on Buffy. Using single features on each body part, the highest accuracy is 90.22% by the LBP feature on torso. Using body part combination, the highest accuracy can be 93.30% by the LBP feature on parts of head, torso, and left upper arm. The SIFT has lower accuracies than the other three features in most cases. So the data fusion is on three representations: RGB, LBP, and HOG. The feature-level fusion achieves the highest accuracy of 96.11% (with head, torso, and right upper arm), which is higher than the decision-level fusion of 94.19% on four body parts. Data fusion improves the accuracy and the feature-level scheme is better than the decision level. Comparing Table 3 with Table 1, the accuracy of automatic gender recognition (96.11%) is lower than the annotation-based (98.04%), but the difference is very small. This tells that our system is pretty robust with high accuracy in automatic gender recognition on Buffy.

Table 4 and Figure 8(b) show the recognition performance on PASCAL. Using single features on each body part, the highest accuracy is 66.29% by the HOG feature on head. Using body part combination, the highest accuracy is 66.14% by the LBP feature on parts of head, torso, and right upper arm. The RGB representation has lower accuracies than the other three features in most cases. So the data fusion is on three representations: LBP, HOG, and SIFT. The decision-level fusion achieves the highest accuracy of 67.74% (with head, torso, and right upper arm), which is higher than the feature-level fusion of 66.44% on four body parts. Data fusion improves the accuracy and the decision-level scheme is better than the feature-level one. Comparing Table 4 with Table 2, the accuracy of automatic gender recognition (67.74%) is lower than the annotation-based (73.82%), but the difference is not very big. This indicates that the proposed approach is pretty robust in automatic gender recognition on PASCAL. On the other hand, the recognition accuracy on PASCAL is much lower than on Buffy; this suggests that the PASCAL database is much more challenging than Buffy.

### 3.3. The PLS Method

As mentioned in previous section, the PLS method can be used for dimensionality reduction and also classification. These two properties are evaluated in our problem separately. To save space, only the results using annotated body parts on PASCAL are shown. Similar performance was observed in automated recognition (not shown here). The experimental results are given in Table 5, where “PLS_1” denotes that the PLS is used just for dimensionality reduction, while “PLS_2” denotes that the PLS is used for both dimensionality reduction and classification, thus avoiding the use of SVMs. In our experiments, the PLS method does not work on SIFT features. One possible reason is that some SIFT features are zero vectors which prohibit the PLS computation. But the PLS can work on other features without any problem. When single feature is used, the recognition accuracy is 70.11% by the approach of HOG + PLS + SVM on three body parts, or 69.41% by the LBP + PLS (no SVM classification) on the same three body parts. When the HOG and LBP features are combined, the PLS gets an accuracy of 70.57% on three body parts. Overall, the results based on PLS are slightly lower than without using it (see Table 2), but the differences are small. Please note that, no matter what the original feature dimensions are (see Table 6), the PLS method efficiently reduces the dimensions to a small number of** 40**. Empirically, we found that using this small number of latent variables is sufficient, even if the original dimension could be as high as 4,355 or 5,360 in Table 6. For time-critical or real-time applications, the PLS method is a good choice to obtain an extremely low dimension and even avoid the use of complex classifiers (e.g., SVMs) in recognition.

### 3.4. A Summary

We have performed comprehensive experiments on gender recognition from unconstrained and articulated human body on two databases, the Buffy and PASCAL. Four different representations have been evaluated on each body part and various combinations of body parts. Two data fusion schemes have been validated. The feature-level fusion scheme is slightly better than the decision-level on Buffy, but slightly worse on PASCAL. The best recognition accuracies achieved are 98.04% (with annotated parts) and 96.11% (automated detection and recognition), respectively, on Buffy, while 73.82% and 67.74%, respectively, on PASCAL. The PLS method efficiently reduces the feature dimensions from a very high number (e.g., 5,360) to an extremely small number of 40.

## 4. Conclusions and Future Work

This paper studies the new problem of gender recognition in unconstrained images of the articulated human body. A gender recognition system has been presented based on upper-body detection, body parts localization, and parts normalization. Through comprehensive empirical studies, we have discovered that the lower arms are not informative; the torso, head, and upper arms can be combined together to recognize gender. And data fusion schemes have advantages over single representations to deliver a more robust solution with good performance. The PLS method can efficiently reduce the dimensionality and classify the patterns. The PASCAL database is more challenging than Buffy, so the future research may study more advanced learning methods to improve the gender recognition accuracy on the PASCAL database.

## Figures and Tables

**Table 1:** Gender recognition accuracies (%) with annotated body parts on Buffy. T: torso, H: head, LUA: left upper arm, RUA: right upper arm, LLA: left lower arm, and RLA: right lower arm. They are also used in other tables and figures.

	RLA	LLA	RUA	LUA	T	H	H + T	H + T + LUA	H + T + RUA	H + T + RUA + LUA
HOG	83.32	85.40	91.26	88.37	90.60	92.23	93.15	92.34	94.23	94.11
LBP	75.58	79.48	94.19	92.14	95.07	94.23	96.12	96.12	96.12	96.12
SIFT	69.39	75.40	85.52	84.28	82.16	92.07	93.18	93.14	93.18	93.18
RGB	81.70	83.29	92.22	89.33	90.41	91.26	**97.12**	96.19	95.26	94.22

F: decision level	85.33	88.33	92.19	94.19	97.12	96.15	95.15	96.12	94.23	96.12
F: feature level	78.67	84.45	96.19	92.22	96.12	95.19	** 98.04**	97.12	98.00	97.12

**Table 2:** Gender recognition accuracies (%) with annotated body parts on PASCAL.

	RLA	LLA	RUA	LUA	T	H	H + T	H + T + LUA	H + T + RUA	H + T + RUA + LUA
HOG	58.90	58.90	62.40	60.25	59.40	67.87	67.99	68.28	71.03	70.93
LBP	58.90	58.90	64.80	63.96	63.78	67.68	67.66	68.90	70.92	**71.62**
SIFT	58.90	58.90	64.16	62.15	59.06	65.49	66.18	68.35	70.07	69.88
RGB	58.90	58.90	58.90	58.90	58.90	58.90	58.90	59.36	59.97	59.09

F: decision level	58.90	58.90	67.43	64.51	64.73	69.55	70.62	70.99	**73.82**	73.42
F: feature level	58.90	58.99	65.30	63.21	64.60	67.83	69.08	71.34	72.30	73.14

**Table 3:** Automated gender recognition accuracies (%) on Buffy.

	RLA	LLA	RUA	LUA	T	H	H + T	H + T + LUA	H + T + RUA	H + T + RUA + LUA
HOG	68.53	71.50	87.18	81.40	90.18	84.44	92.29	93.26	92.10	92.26
LBP	65.94	73.44	86.18	83.63	90.22	80.62	92.34	** 93.30**	91.22	92.11
SIFT	66.84	65.91	78.74	73.67	79.24	79.47	83.47	87.40	87.40	89.37
RGB	80.39	69.46	93.18	84.33	86.48	89.22	91.33	92.30	89.37	92.33

F: decision level	70.76	74.36	92.99	81.43	92.26	86.33	95.23	91.26	93.26	94.19
F: feature level	67.95	74.44	89.99	84.60	92.18	84.52	93.26	93.15	** 96.11**	94.26

**Table 4:** Automated gender recognition accuracies (%) on PASCAL.

	RLA	LLA	RUA	LUA	T	H	H + T	H + T + LUA	H + T + RUA	H + T + RUA + LUA
HOG	58.90	58.90	61.63	63.98	58.90	66.29	60.90	62.53	63.39	63.79
LBP	58.90	58.90	64.28	59.93	58.90	63.89	63.90	63.75	** 66.14**	65.84
SIFT	59.37	61.11	59.60	60.55	60.62	62.33	63.05	64.33	65.32	65.04
RGB	58.90	58.90	58.90	58.90	58.66	58.90	58.90	58.90	58.90	58.90

F: decision level	58.90	58.90	64.21	63.07	58.90	64.93	65.69	64.78	** 67.74**	66.72
F: feature level	58.90	60.81	63.91	64.00	59.40	64.34	63.21	65.52	66.26	66.44

**Table 5:** Gender recognition accuracies (%) using the PLS method for dimensionality reduction (denoted by PLS_1) or both dimensionality reduction and classification (denoted by PLS_2) on PASCAL data with annotated body parts.

	RLA	LLA	RUA	LUA	T	H	H + T	H + T + LUA	H + T + RUA	H + T + RUA + LUA
HOG_PLS_1	58.90	58.90	62.38	60.28	58.90	65.23	65.21	65.97	**70.11**	69.56
LBP_PLS_1	58.90	58.90	62.30	60.06	62.59	65.25	67.28	67.36	69.93	69.01
RGB_PLS_1	57.37	58.91	58.70	58.12	57.73	58.52	58.90	58.90	58.20	58.90

HOG_PLS_2	52.82	48.89	56.85	56.53	57.02	62.04	59.64	58.05	58.59	58.73
LBP_PLS_2	50.98	50.69	54.95	53.00	57.84	63.09	65.40	66.69	**69.41**	68.72
RGB_PLS_2	53.27	51.64	58.58	50.66	58.27	56.04	52.66	53.97	54.10	53.84

HOG + LBP_1	58.90	58.90	63.10	61.59	63.19	67.09	66.40	68.14	**70.57**	69.08
HOG + LBP_2	48.07	50.50	55.70	54.08	60.25	66.11	67.67	67.59	69.80	69.57

**Table 6:** Feature dimensions with various setups on PASCAL. No matter what the original dimensions are, the PLS method can efficiently reduce each number to a small value of **40**, which is very useful for real-time applications.

	RLA	LLA	RUA	LUA	T	H	H + T	H + T + LUA	H + T + RUA	H + T + RUA + LUA
HOG	120	120	120	120	200	200	400	520	520	640
LBP	885	885	885	885	1475	1475	2950	3835	3835	4720
SIFT	1920	1920	1920	1920	3200	3200	6400	8320	8320	10240
RGB	96	96	96	96	96	96	192	288	288	384
HOG + LBP	1005	1005	1005	1005	1675	1675	3350	4355	4355	5360
